# Regulation of Skeletal Muscle DRP-1 and FIS-1 Protein Expression by IL-6 Signaling

**DOI:** 10.1155/2019/8908457

**Published:** 2019-02-21

**Authors:** Dennis K. Fix, Brandon N. VanderVeen, Brittany R. Counts, James A. Carson

**Affiliations:** ^1^Integrative Muscle Biology Laboratory, Division of Applied Physiology, Department of Exercise Science, University of South Carolina, Columbia, SC, USA; ^2^College of Health Professions, Department of Physical Therapy, University of Tennessee Health Sciences Center, Memphis, TN 38163, USA

## Abstract

IL-6 signals through the ubiquitously expressed glycoprotein 130 (gp130) transmembrane protein to activate intracellular signaling that includes signal transducer and activator of transcription 3 (STAT3) and extracellular signal-regulated kinase 1/2 (ERK1/2). Dynamin-1-like protein (DRP-1) and mitochondrial fission 1 protein (FIS-1) are key proteins in the process of mitochondrial fission and have emerged as IL-6-sensitive targets. The purpose of this study was to examine the regulation of DRP-1 and FIS-1 expression by IL-6 and gp130 signaling in myotubes and skeletal muscle. Fully differentiated C2C12 myotubes were treated with 100 ng of IL-6 for 24 hours in the presence of gp130siRNA, C188-9 (STAT3 inhibitor), or PD98059 (ERK1/2 inhibitor). Male C57BL/6 (B6) and muscle-specific gp130 knockout mice (KO) had IL-6 systemically overexpressed for 2 weeks by transient transfection with 50 ng of an IL-6-expressing or control plasmid in the quadriceps muscles, and the tibialis anterior muscle was analyzed to determine systemic effects of IL-6. IL-6 induced DRP-1 and FIS-1 expression in myotubes 124% and 82% (*p* = .001) and in skeletal muscle 97% and 187% (*p* = .001). Myotube gp130 knockdown suppressed the IL-6 induction of DRP-1 68% (*p* = .002) and FIS-1 65% (*p* = .001). Muscle KO suppressed the IL-6 induction of DRP-1 220% (*p* = .001) and FIS-1 121% (*p* = .001). ERK1/2 inhibition suppressed the IL-6 induction of DRP-1 59% (*p* = .0003) and FIS-1 102% (*p* = .0001) in myotubes, while there was no effect of STAT3 inhibition. We report that chronically elevated IL-6 can directly induce DRP-1 and FIS-1 expression through gp130 signaling in cultured myotubes and skeletal muscle. Furthermore, ERK 1/2 signaling is necessary for the IL-6 induction of DRP-1 and FIS-1 expression in myotubes.

## 1. Introduction

Chronic inflammation is a hallmark of many illnesses, `including cancer, diabetes, and cardiovascular disease. Furthermore, skeletal muscle glucose metabolism and mass regulation are disrupted by these conditions [1, 2]. The interleukin-6 (IL-6) cytokine family has been investigated extensively as a critical driver of inflammation during chronic disease and is an established effector of skeletal muscle dysfunction [2–6]. IL-6 is a pleiotropic cytokine capable of serving as both pro- and anti-inflammatory. Classically, intracellular IL-6 signaling is induced through binding with a specific IL-6 cytokine receptor that dimerizes with glycoprotein 130 (gp130), a ubiquitously expressed transmembrane protein [7–9]. IL-6 signaling can also be initiated through trans-signaling, whereby IL-6 binds to the soluble form of the IL-6 receptor to initiate cellular signaling through interaction with gp130 on the cell membrane [10]. IL-6 is capable of inducing several intracellular signaling pathways that can regulate skeletal muscle mass and metabolism. IL-6 can induce skeletal muscle signal transducer and activator of transcription 3 (STAT3) and extracellular regulated kinase (ERK1/2) in several preclinical cancer cachexia models [2, 3, 11–15]. While IL-6 signaling has established a role in the regulation of muscle mass and metabolism; a role for regulating skeletal muscle mitochondria homeostasis has not been clearly established.

Skeletal muscle mitochondria are essential for maintaining metabolic plasticity and function [4, 16, 17]. Mitochondrial quality control encompasses the biogenesis, turnover (mitophagy), and remodeling (dynamics) of mitochondria [18–22]. Chronic disease can disrupt all of these skeletal muscle mitochondria quality control components, and they have been connected to the skeletal muscle proteostasis that occurs with these conditions [18]. Mitochondrial remodeling (dynamics) is a process that consists of constant fission and fusion of mitochondria in response to metabolic stressors [19, 21, 23]. Fission is controlled by GTPase cytosolic dynamin-related protein-1 (DRP-1), which will translocate to the outer mitochondrial membrane and develop active fission sites [17, 23–26]. Fission protein 1 (FIS-1) recruits DRP-1 to the mitochondria [21, 22]. The acceleration of fission can result in the isolation of mitochondria from the network and reduced ATP efficiency, resulting in turnover or apoptosis [17, 22, 27]. Altered mitochondrial fission has been linked to skeletal muscle mass regulation [19, 21, 28]. Since accelerated fission can result in muscle metabolic dysfunction, and the attenuation of fission can result in muscle atrophy, mitochondrial remodeling processes appear necessary for overall muscle homeostasis [18, 29].

STAT3 and ERK1/2 are downstream effectors of IL-6 that have established roles in skeletal skeletal muscle mass regulation. STAT3 is a widely investigated downstream effector of IL-6 in skeletal muscle [10, 14, 30–34], and chronic STAT3 activation can drive muscle atrophy through accelerated protein degradation [3, 4, 33, 35]. STAT3 can target mitochondrial function through complex I suppression [36]. ERK1/2 signaling is also an established regulator of skeletal muscle dysfunction during cancer cachexia, chemotherapy, and exercise [37–41]. ERK1/2 activation coincides mitochondrial content loss and biogenesis suppression during chemotherapy-induced cachexia [41]. Furthermore, ERK1/2 activation can promote DRP-1-mediated fission in MEF cells [42], which has not been established in skeletal muscle. While STAT3 and ERK1/2 are well-characterized signaling pathways in skeletal muscle, their regulation of skeletal muscle mitochondrial fission warrants further investigation.

Chronically elevated circulating IL-6 has been reported in cancer patients and preclinical models of cancer cachexia. Muscle wasting is also often associated with increased skeletal muscle expression of mitochondrial fission regulating proteins [4, 20, 22, 24]. Furthermore, increased FIS-1 expression in cachectic skeletal muscle from tumor-bearing mice is dependent on IL-6; tumor-bearing mice treated with an IL-6 receptor antibody demonstrated an attenuation of muscle STAT3 and FIS-1 expression [15]. Our laboratory has also demonstrated that chronically elevated IL-6 can increase STAT3 activation and FIS-1 expression in myotubes and skeletal muscle independent of a cancer environment [15]. While these findings suggest that circulating IL-6 can regulate skeletal muscle mitochondrial fission [4, 15], the intracellular signaling that links IL-6 to increased mitochondrial fission requires further investigation. Therefore, the purpose of this study was to examine the regulation of DRP-1 and FIS-1 expression by IL-6 and gp130 signaling in skeletal muscle. Additionally, we investigated the role of intracellular STAT3 and ERK 1/2 activation in myotubes. We report that chronically elevated IL-6 can directly induce DRP-1 and FIS-1 expression through gp130 signaling in cultured myotubes and skeletal muscle. Furthermore, ERK 1/2 signaling is necessary for the IL-6 induction of DRP-1 and FIS-1 expression in myotubes.

## 2. Methods

### 2.1. Animals

Male C57BL/6 (Bar Harbor, ME, USA) were bred at the University of South Carolina's animal resource facility, as previously described [7, 43, 44]. Male mice on a C57BL/6 background were bred with gp130 floxed mice provided by Dr. Colin Stewart's laboratory [Laboratory of Cancer and Developmental Biology, National Cancer Institute, US National Institutes of Health (NIH), Frederick, MD, USA] in collaboration with Dr. Lothar Hennighausen (Laboratory of Genetics and Physiology, National Institute of Diabetes and Digestive and Kidney Diseases, NIH, Bethesda, MD, USA) and crossed with a myosin light chain cre promoter, as previously published [7, 43, 44]. All mice were group-housed (five to a cage) and were sacrificed at 12 weeks of age. All experiments and methods performed were done in accordance with relevant guidelines and regulations at the University of South Carolina. The Institutional Animal Care and Use Committee at the University of South Carolina approved all experiments.

### 2.2. IL-6 Overexpression

To increase circulating IL-6 levels, a total of 32 mice, 16 WT and 16 KO, were divided into two subgroups (vector; *n* = 16 and + IL-6; *n* = 16) for IL-6 overexpression experiments. *In viv*o intramuscular electroporation of an IL-6 plasmid into the quadriceps muscle was used to increase circulating IL-6 levels in mice, as previously described [15]. The quadriceps muscle was used as a vessel to produce IL-6 and secrete it into circulation and was not used for any analyses in this study. In order to test the systemic effects of our IL-6 treatment, the tibialis anterior muscle was used for analysis in this study and was not subjected to electroporation or any kind of electrical stimulation. Briefly, mice were anesthetized with a 2% mixture of isoflurane and oxygen (1 L/minute). The leg was shaved, and a small incision was made over the quadriceps muscle. Fat was dissected away from the quadriceps muscle, which was then injected with 50 *μ*g of the IL-6 plasmid driven by the CMV promoter or empty control vector [44]. A series of eight 50 ms, 100 V pulses was used to promote uptake of the plasmid into myofibers, and then the incision was closed with a wound clip [5, 44]. Both vector control and IL-6 groups received the appropriate plasmid starting at 10 weeks of age; at 11 weeks, the mice underwent the same procedure on the opposite quadriceps muscle in order to maintain elevated IL-6 levels over a two-week period. All mice were euthanized 7 days after their second electroporation.

### 2.3. C2C12 Myotube Cell Culture

C2C12 myoblasts (American Type Culture Collection, Manassas, VA, USA) were cultured in DMEM, supplemented with 10% FBS, 50 U/mL penicillin, and 50 *μ*g/mL streptomycin [7]. At >90% confluency, C2C12 myoblasts were incubated in a differentiation medium (DMEM supplemented with 2% heat-inactivated horse serum, 50 U/mL penicillin, and 50 *μ*g/mL streptomycin) for 72 h to induce differentiation into myotubes, and experiments were performed. Each experiment was replicated, and all analyses included six replicates per control and treatment group.

### 2.4. C2C12 IL-6 Cytokine Treatment

After a 72 h differentiation period, IL-6 (Sigma, St. Louis, MO, USA) was added to serum-free DMEM and incubated for 24 h. Cells were harvested by washing with ice-cold PBS and then scraped in ice-cold lysis buffer (50 mM Tris, 150 mM NaCl, 1 mM EDTA, 1% Triton X-100, 0.1% SDS, 0.5% sodium deoxycholate, 5 mM NaF, 1 mM *β*-glycerophosphate, 1 mM NaVO_3_, and 1/200 protease inhibitor cocktail (Sigma, P8340), pH 8.0). After sonication, cell debris was removed by centrifugation, and the supernatant was stored at −80°C. Protein concentrations were measured by the Bradford assay (Bio-Rad, Hercules, CA, USA), and the samples were used for Western blot analysis. Each experiment was replicated, and all analyses included six replicates per control and treatment groups.

### 2.5. RNA Interference

C2C12 myotubes were transfected with scramble siRNA (GE Dharmacon, Lafayette, CO, USA) or gp130 siRNA (Santa Cruz Biotech, Santa Cruz, CA, USA) 3 days post-differentiation using DharmaFECT 3 transfection reagent (GE Dharmacon, Lafayette, CO, USA), according to the manufacturer's instructions and as previously described [43]. Briefly, siRNA and transfection reagent were separately diluted in serum-free and antibiotics-free DMEM and incubated at room temperature for 5 min [43]. The diluted transfection reagent was then added to the siRNA mixture and allowed to complex with siRNA for 20 min. siRNA-transfection reagent complexes were then added to the antibiotics-free differentiation medium, and myotubes were incubated for 24 hours in a transfection-containing medium (100 nM siRNA concentration). Transfection efficiency was validated by cotransfecting 20 nM (final concentration) of siGLO RISC-Free Control siRNA (GE Dharmacon, Lafayette, CO, USA). Fluorescence was visualized by a Cy3 filter to determine the transfection efficiency. Validated transfected myotubes were collected for protein analysis.

### 2.6. C2C12 Myotube STAT3 and ERK 1/2 Inhibition

Following differentiation, a STAT3 inhibitor, C188-9 (10 *μ*M) (BioVision, Milpitas, CA, USA), was added to a culture medium for a duration of 2 hours [35]. Following 2 hours, myotubes were treated with or without IL-6 for a duration of 24 hours and collected for western blot analysis [35]. To inhibit ERK1/2 signaling, myotubes were treated with PD98059 (20 *μ*M, Cell Signaling Technology, Danvers MA) with or without the presence of IL-6 (100 ng) for a duration of 24 hours. Following 24 hours of incubation, myotubes were collected for western blot analysis. Each experiment was replicated, and all analyses included six replicates per control and treatment groups.

### 2.7. Statistics

Utilizing previous experiments from our laboratory, we conducted a power analysis to determine the sample size needed to observe statistical significance [15]. Power (1 − *β*) was set to 0.8, and error of probability (*α*) was set at 0.05. Based on previous results using a similar treatment and protein of interest (FIS-1) [15], to achieve significance for an estimated 30 ± 10% difference between groups, a sample size of 8 animals is needed per group. Results are reported as means ± standard error of the mean. Analysis was completed using either a standard one-way analysis of variance (ANOVA), two-way ANOVA, or Student's *t*-test, as appropriate. Post hoc analyses were performed with the Tukey's multiple comparison test when appropriate. Significance was set at *p* < 0.05. Statistical analysis was performed using GraphPad Prism version 7.0 (La Jolla, CA, USA).

## 3. Results

### 3.1. IL-6 Induction of Inflammatory Signaling, DRP-1, and FIS-1 in C2C12 Myotubes

**Figure 1:**
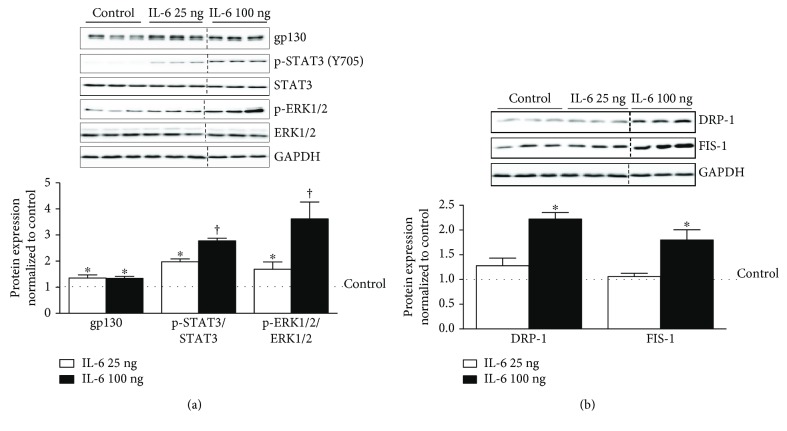
IL-6 activation of gp130 signaling and mitochondrial fission in C2C12 myotubes. Fully differentiated C2C12 myotubes were treated with either 25 or 100 ng of IL-6 for a duration of 24 hours. (a). Upper: representative immunoblot of gp130, p-STAT3 (Y705), STAT3, p-ERK1/2, ERK, and GAPDH. Lower: quantification of above immunoblots. Dashed line indicates blot was cropped for representative purposes. (b). Upper: representative immunoblot DRP-1, FIS-1, and GAPDH. Lower: quantification of above immunoblots. All values are reported as means ± standard error. Six total replicates were used for all analysis. All values were normalized to control (dashed line on graph). Analysis was conducted using a standard one-way analysis of variance (ANOVA). Statistical significance was set at *p* < 0.05. ^∗^Statistically different from control. †Statistically different to all groups.

In order to examine and validate our IL-6 signaling pathway, fully differentiated myotubes were treated with a low and high dose (25 ng or 100 ng) of IL-6 for a duration of 24 hours and immediate downstream targets were analyzed. IL-6 treatment induced gp130 protein expression by 38% and was independent of IL-6 dose used (Figure 1(a)). The activation of STAT3 Y705 was induced 101% by 25 ng (*p* = .001) and further induced to 181% by 100 ng of IL-6 (*p* = .001), when compared to control (Figure 1(a)). ERK 1/2 activation followed a similar trend as STAT3, being induced by 73% with 25 ng and further induced by 266% with 100 ng of IL-6, when compared to control (Figure 1(a)). Next, we examined the activation of mitochondrial fission proteins DRP-1 and FIS-1. DRP-1 and FIS-1 were not altered with 25 ng of IL-6 but were induced by 124% and 82% above the control following 100 ng of IL-6 treatment (Figure 1(b)).

### 3.2. gp130 Regulation of DRP-1 and FIS-1 in IL-6-Treated C2C12 Myotubes

**Figure 2:**
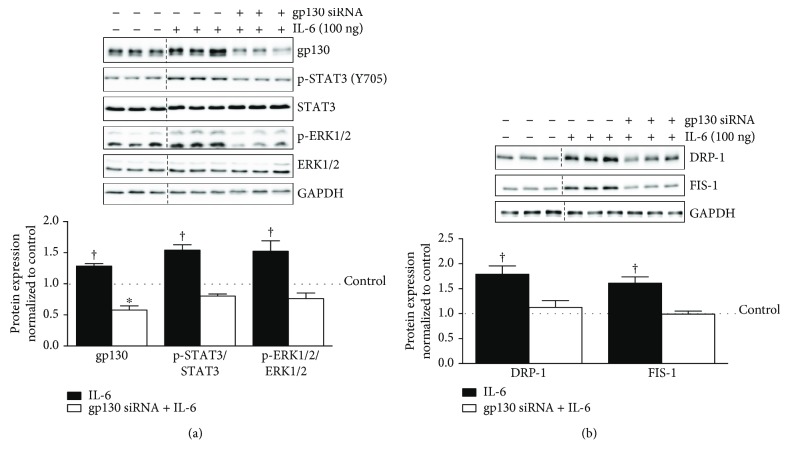
gp130 regulation of mitochondrial fission proteins DRP-1 and FIS-1 in IL-6-treated C2C12 myotubes. We next examined the role of immediate downstream IL-6 target gp130 in the regulation of IL-6 signaling and mitochondrial fission proteins DRP-1 and FIS-1 in fully differentiated C2C12 myotubes. (a). Upper: representative immunoblot of gp130, p-STAT3 (Y705), STAT3, p-ERK1/2, ERK, and GAPDH in myotubes treated with 100 ng of IL-6 or the combination of IL-6 and gp130 siRNA. Lower: quantification of above immunoblots. Dashed line indicates blot was cropped for representative purposes. (b). Upper: representative immunoblot DRP-1, FIS-1, and GAPDH in myotubes treated with 100 ng of IL-6 or the combination of IL-6 and gp130 siRNA. Lower: quantification of above immunoblots. All values are reported as means ± standard error. Six total replicates were used for all analysis. All values were normalized to control (dashed line on graph). Analysis was conducted using a standard one-way analysis of variance (ANOVA). Statistical significance was set at *p* < 0.05. ^∗^Statistically different from control. †Statistically different to all groups.

To examine gp130's role in mitochondrial fission, siRNA was used to knock down gp130 expression in C2C12 myotubes. Myotubes were treated with either a scrambled siRNA (Control) or a gp130 siRNA. The protein expression of gp130 was suppressed by 30% in the basal state with siRNA treatment (Supplemental Figure 1A). Moreover, gp130 siRNA treatment suppressed STAT3 and ERK1/2, while inducing DRP-1 and FIS-1 in the basal state (Supplemental Figure 1B). To mechanistically examine the role of gp130 in the IL-6 induction of DRP-1 and FIS-1, C2C12 myotubes were treated with either a scrambled siRNA (Control) or a gp130 siRNA in the presence of 100 ng of IL-6. IL-6 induced gp130 protein expression by 30% compared to control myotubes and was significantly different compared to all groups (*p* = .002). The IL-6 induction of gp130 was blocked with gp130 siRNA treatment 71% (*p* = .0004) and was actually 41% lower than control-treated myotubes (*p* = .0002) (Figure 2(a)). gp130 siRNA suppressed STAT3 and ERK 1/2 phosphorylation 75% (*p* = .004) in the presence of IL-6 (Figure 2(a)). The IL-6 induction of DRP-1 was suppressed by 68% (*p* = .002) and FIS-1 by 65% (*p* = .001) by gp130 siRNA treatment (Figure 2(b)). Collectively, these results demonstrate a role for IL-6-induced gp130 signaling in the regulation of DRP-1 and FIS-1 expression in C2C12 myotubes.

### 3.3. STAT3 Regulation of DRP-1 and FIS-1 in IL-6-Treated C2C12 Myotubes

**Figure 3:**
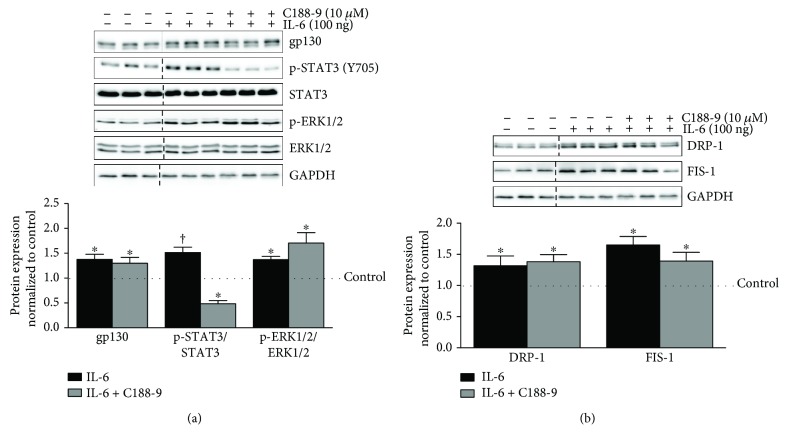
STAT3 regulation of mitochondrial fission proteins DRP-1 and FIS-1 in IL-6-treated C2C12 myotubes. We moved further downstream of gp130 and examined the role of STAT3 in the regulation of IL-6 signaling and mitochondrial fission proteins DRP-1 and FIS-1 in IL-6-treated C2C12 myotubes. (a). Upper: representative immunoblot of gp130, p-STAT3 (Y705), STAT3, p-ERK1/2, ERK, and GAPDH in myotubes treated with 100 ng of IL-6 or the combination of IL-6 and C188-9 (STAT3 small molecule inhibitor). Lower: quantification of above immunoblots. Dashed line indicates blot was cropped for representative purposes. (b). Upper: representative immunoblot DRP-1, FIS-1, and GAPDH in myotubes treated with 100 ng of IL-6 or the combination of IL-6 and C188-9. Lower: quantification of above immunoblots. All values are reported as means ± standard error. Six total replicates were used for all analysis. All values were normalized to control (dashed line on graph). Analysis was conducted using a standard one-way analysis of variance (ANOVA). Statistical significance was set at *p* < 0.05. ^∗^Statistically different from control. †Statistically different to all groups.

We continued our examination of IL-6 signaling by inhibiting STAT3 phosphorylation in differentiated myotubes treated with 100 ng of IL-6. STAT inhibitor C188-9 suppressed basal STAT3 phosphorylation by 66% without altering basal ERK 1/2 phosphorylation (Supplemental Figure 1C). Basal STAT3 inhibition did not alter DRP-1 and FIS-1 (Supplemental Figure 1D). The IL-6 induction of STAT3 (Y705) phosphorylation was suppressed by 103% (*p* = .0003) with C188-9 treatment and was 50% lower (*p* = .01) than that of control myotubes (Figure 3(a)). C188-9 did not alter the IL-6 induction of gp130 expression or ERK1/2 phosphorylation (Figure 3(a)). We next examined mitochondrial fission proteins DRP-1 and FIS-1 in the presence of high IL-6 with C188-9. STAT3 inhibition by C188-9 did not alter the IL-6 induction of DRP-1 or FIS-1, suggesting that the regulation of these proteins by IL-6 occurs via a STAT3-independent mechanism (Figure 3(b)).

### 3.4. ERK 1/2 Regulation of Mitochondrial Fission Proteins DRP-1 and FIS-1 in IL-6-Treated C2C12 Myotubes

**Figure 4:**
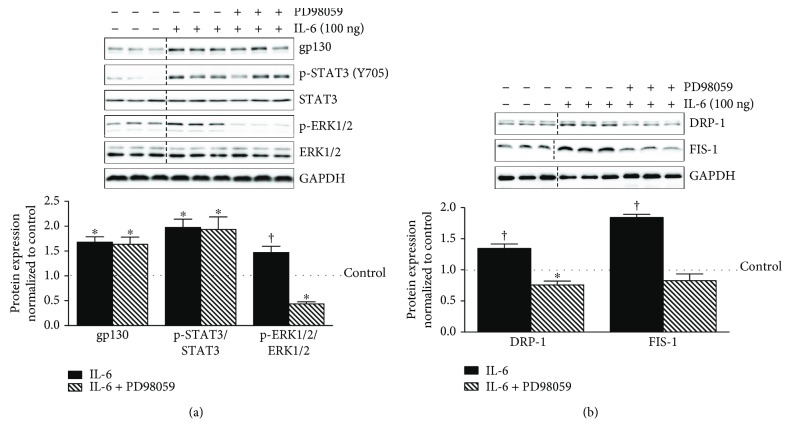
ERK1/2 regulation of mitochondrial fission proteins DRP-1 and FIS-1 in IL-6-treated C2C12 myotubes. Continuing our mechanistic investigation of IL-6 signaling, we next examined the role of ERK1/2 in the regulation of mitochondrial fission proteins DRP-1 and FIS-1 in IL-6-treated C2C12 myotubes. (a). Upper: representative immunoblot of gp130, p-STAT3 (Y705), STAT3, p-ERK1/2, ERK, and GAPDH in myotubes treated with 100 ng of IL-6 or the combination of IL-6 and PD98059. Lower: quantification of above immunoblots. Dashed line indicates blot was cropped for representative purposes. (b). Upper: representative immunoblot DRP-1, FIS-1, and GAPDH in myotubes treated with 100 ng of IL-6 or the combination of IL-6 and PD98059. Lower: quantification of above immunoblots. All values are reported as means ± standard error. Six total replicates were used for all analysis. All values were normalized to control (dashed line on graph). Analysis was conducted using a standard one-way analysis of variance (ANOVA). Statistical significance was set at *p* < 0.05. ^∗^Statistically different from control. †Statistically different to all groups.

We next examined ERK 1/2 signaling using inhibitor PD98059. ERK 1/2 inhibition suppressed basal ERK 1/2 phosphorylation 44%, DRP-1 52%, and FIS-1 47% (Supplemental Figures 1E and F). IL-6 treatment induced ERK 1/2 phosphorylation 49% above control level (*p* = .01), which was reduced by 103% (*p* = .0004) by PD98059 (Figure 4(a)). PD98059 did not alter the IL-6 induction of gp130 or STAT3 (Figure 4(a)). The IL-6 induction of DRP-1 and FIS-1 protein expression was reduced by 59% (*p* = .0003) and 102% (*p* = .0001) by PD98059 treatment (Figure 4(b)). These results collectively demonstrate that the IL-6 regulation of DRP-1 and FIS-1 protein expression occurs through a gp130/ERK1/2 signaling axis.

### 3.5. In Vivo Systemic IL-6 Overexpression Induces Muscle gp130 Signaling, DRP-1, and FIS-1 In Vivo

**Figure 5:**
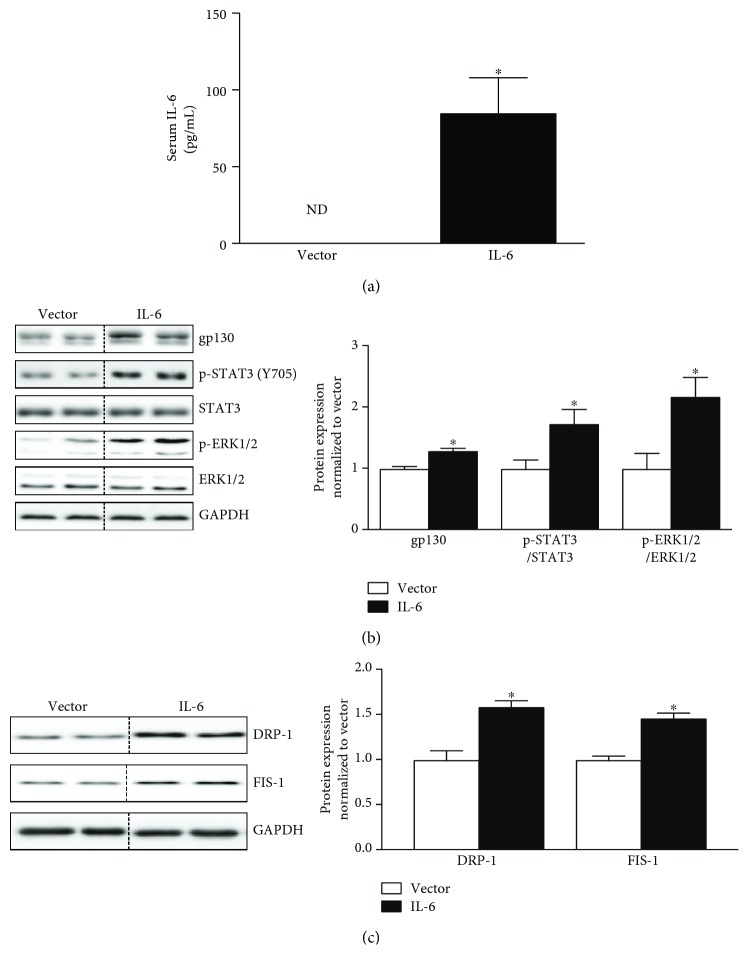
Systemic IL-6 overexpression induces muscle gp130 signaling and mitochondrial fission proteins DRP-1 and FIS-1 *in vivo*. We next examined if systemic IL-6 overexpression is capable of inducing muscle gp130 signaling and mitochondrial fission proteins *in vivo*. (a). Circulating serum IL-6 levels in vector and IL-6-treated mice following 2 weeks of IL-6 overexpression. (b). Left: representative immunoblot of gp130, p-STAT3 (Y705), STAT3, p-ERK1/2, ERK, and GAPDH in tibialis anterior muscle of C57BL/6 mice treated with either vector or IL-6. Right: quantification of immunoblots. Dashed line indicates blot was cropped for representative purposes. (c). Left: representative immunoblot DRP-1, FIS-1, and GAPDH in tibialis anterior muscle of C57BL/6 mice treated with either vector or IL-6. Right: quantification of immunoblots. All values are reported as means ± standard error. *N* = 8 mice per treatment group. All values were normalized to Vector. Analysis was conducted using an un-paired students t-test. Statistical significance was set at *p* < 0.05. ^∗^statistically different from Control.

IL-6 was systemically overexpressed in vivo through electroporation of the quadriceps muscle with either 50 *μ*g of IL-6 overexpression plasmid or control plasmid in 10-week-old C57BL/6 mice once a week for two weeks, alternating the quadriceps muscle electroporated. Serum IL-6 was elevated following 2 weeks of treatment (Figure 5(a)). Tibialis anterior muscle gp130 protein expression was induced by 31% (*p* = .001) above vector control. Immediate downstream targets STAT3 (Y705) and p-ERK1/2 (Figure 5(b)) were induced by 78% (*p* = .02) and 101% (*p* = .01) with 2 weeks of systemic IL-6 overexpression. Similar to our *in vitro* analysis, the induction of gp130 signaling was also accompanied by a 51% induction of DRP-1 (*p* = .004) and a 48% induction of FIS-1 (*p* = .001) in the tibialis anterior muscle (Figure 5(c)). These results suggest that two weeks of elevated circulating IL-6 induces skeletal muscle mitochondrial fission proteins DRP-1 and FIS-1 *in-vivo*.

### 3.6. IL-6 Regulation of DRP-1 and FIS-1 in gp130 KO Mice

**Figure 6:**
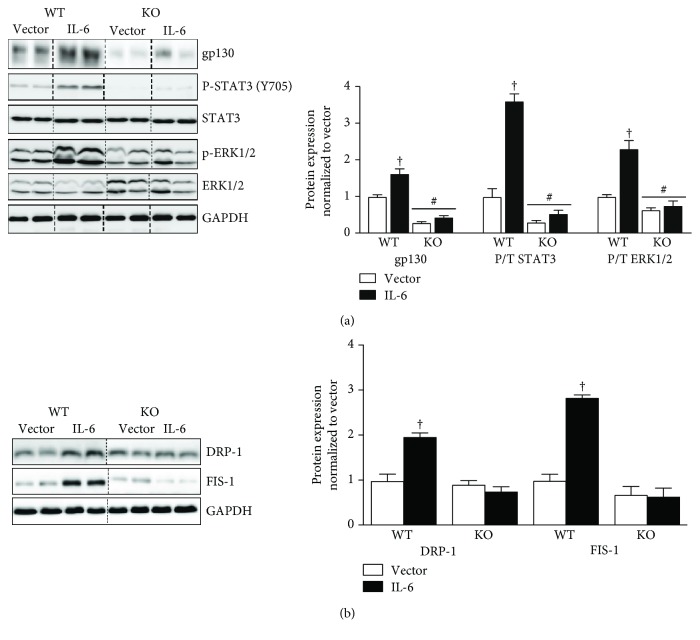
IL-6 regulation of gp130 signaling and mitochondrial fission proteins DRP-1 and FIS-1 *in vivo* in muscle gp130 KO mice. We examined the IL-6 regulation of gp130 and mitochondrial fission proteins DRP-1 and FIS-1 in muscle gp130 KO mice. (a). Left: representative immunoblot of gp130, p-STAT3, STAT3, p-ERK1/2, ERK1/2, and GAPDH in tibialis anterior muscle of WT and KO mice treated with either vector or IL-6. Right: quantification of immunoblot. Dashed line indicates blot was cropped for representative purposes. (b). Left: representative immunoblot DRP-1, FIS-1, and GAPDH in tibialis anterior muscle of WT or KO mice treated with either vector or IL-6. Right: quantification of immunoblots. All values are reported as means ± standard error. *N* = 8 mice per treatment group. All values were normalized to vector. Analysis was conducted using a two-way analysis of variance (ANOVA). Statistical significance was set at *p* < 0.05. †Statistically different to all groups. ^#^Statistically different to WT vector.

We next examined if systemic IL-6 directly regulated DRP-1 and FIS-1 expression in the skeletal muscle through gp130 signaling using a muscle-specific gp130 knockout mouse. Muscle gp130 loss suppressed basal gp130 expression 73%, STAT3 (Y705) phosphorylation 81%, and ERK1/2 phosphorylation 48% (*p* = .0001 for all proteins). The IL-6 induction of tibialis anterior muscle gp130 expression, STAT3 (Y705) phosphorylation, and ERK1/2 phosphorylation were blocked by muscle gp130 loss (Figure 6(a)). Similar to our *in vitro* results, IL-6 induced DRP-1 97% (*p* = .001) and FIS-1 187% (*p* = .001) in the tibialis anterior muscle of the control mice. Muscle gp130 loss was sufficient to suppress the IL-6 induction of DRP-1 220% and FIS-1 121%, (*p* = .0001) in the tibialis anterior muscle (Figure 6(b)). These results demonstrate that systemic IL-6 directly regulates DRP-1 and FIS-1 expression through muscle gp130 signaling.

## 4. Discussion

Mitochondria organelles play the critical role of maintaining skeletal muscle energy balance in [22, 26, 45], and chronic inflammation is a recognized mediator of mitochondrial dysfunction [5, 19, 21, 46, 47]. Chronically elevated systemic IL-6 can alter muscle mitochondrial morphology and negatively regulate oxidative genes [36, 46–48]. While the IL-6 family of cytokines has been associated with the regulation of muscle mitochondrial quality control [7, 43], the direct regulation of systemic IL-6 on intracellular pathways that regulate these mitochondrial processes has not been firmly established. IL-6 could exert an indirect effect on skeletal muscle homeostasis through immune signaling or metabolic processes in other tissues [2, 8]. Our results suggest that IL-6 can directly regulate the expression of skeletal muscle mitochondrial fission proteins DRP-1 and FIS-1. We report the novel finding that suppressing gp130 both *in vitro* and *in vivo* inhibits the IL-6 induction of DRP-1 and FIS-1. Additionally, we provide evidence that ERK1/2 signaling in C2C12 myotubes is sufficient to block the IL-6 induction of DRP-1, while STAT3 signaling was not necessarily the IL-6 induction of DRP-1 and FIS-1 *in vitro*. Collectively, we demonstrate that IL-6 can directly regulate muscle DRP-1 and FIS-1 expression through gp130 signaling, involving ERK1/2 activation. However, further work is needed to establish a role for ERK 1/2 signaling in skeletal muscle.

The mitochondrial fission process in skeletal muscle is a critical component of mitochondrial quality control regulation [15, 18, 20, 22, 24, 25, 43]. FIS-1 and DRP-1 proteins are critical regulators of skeletal muscle fission regulation by either exercise or chronic inflammation [20]. The metabolic consequences of accelerated fission have been demonstrated by selective targeting of either DRP-1 or FIS-1 proteins. However, fission inhibition can also be detrimental for muscle metabolic quality [17, 20, 25]. While FIS-1 and DRP-1 have been associated with the fission process, the mechanisms regulating these proteins are just beginning to be understood [4, 21]. Inflammation functions as an effector of mitochondrial fission through DRP-1 and FIS-1 [15, 19, 21]. Proinflammatory cytokines such as IL-6, TNF-*α*, and TGF-*β* have been widely investigated for their roles in chronic inflammation and have now been implicated in disrupting mitochondrial quality control through the aberrant regulation of fission [2, 49]. Circulating IL-6 is a driver of muscle wasting in the *Apc^Min/+^* mouse model of cancer cachexia, and cachexia is accompanied by altered mitochondrial dynamics and increased FIS-1 protein expression [15]. The administration of an IL-6 receptor antibody systemically can attenuate muscle mass loss and suppressed muscle FIS-1 expression in tumor-bearing mice [15]. As previously reported, our results confirm that IL-6 is sufficient to induce muscle FIS-1 expression in the absence of a cancer environment *in vivo*. We have extended these findings to demonstrate that DRP-1 expression in also sensitive to circulating IL-6, which provides additional evidence for muscle mitochondrial fission processes being targeted by IL-6. Furthermore, we provide *in vitro* and *in vivo* evidence that the gp130 transmembrane protein in myotubes and skeletal muscle fibers is necessary for the IL-6 induction of DRP-1 and FIS-1 protein expression. Further work is warranted to determine if the suppression of specific gp130 downstream effectors could benefit aberrant skeletal muscle mitochondrial fission induced by chronic disease and cancer.

There is evidence that aberrant mitochondrial fission can negatively impact muscle mitochondrial quality control and lead to disrupted muscle metabolic homeostasis [19, 20, 29, 50]. While STAT3 activation is classically associated with IL-6 signaling [10], ERK1/2 is also activated through IL-6/gp130 signaling [29, 38, 42, 47, 51, 52]. ERK1/2 is commonly viewed as a stress response signal and has been investigated for a regulatory role in inflammation caused by cancer and chemotherapeutics [38, 47]. The IL-6 activation of ERK1/2 can suppresses PGC-1*α* gene expression in skeletal muscle of aged rats and in C2C12 myotubes [53]. Mitochondrial depletion during chemotherapy-related cachexia has also been associated with ERK1/2 signaling [47]. In the current study, IL-6 induced ERK1/2 phosphorylation in skeletal muscle and cultured myotubes. Knockdown of gp130 using either siRNA or muscle specific knockout inhibited the IL-6 induction of ERK1/2 phosphorylation and the subsequent induction of DRP-1 and FIS-1. Specifically, ERK1/2 inhibition in cultured myotubes attenuated the IL-6 induction of DRP-1 and FIS-1 expression. While these findings suggest that IL-6 regulates muscle DRP-1 and FIS-1 expression through a gp130/ERK1/2 signaling axis *in vitro*, further examination is needed to link gp130/ERK1/2 signaling to the mitochondrial fission process in skeletal muscle. Furthermore, it is interesting to speculate on whether this induction of fission is a driver of metabolic dysfunction or if this is a compensatory mechanism for other muscle homeostatic disruptions initiated by inflammation. Additional experimentation needs to examine if altered fission directly leads to muscle metabolic and functional decrements that accompany chronic disease.

Small molecule inhibitors have demonstrated a role for STAT3 in the regulation of muscle mass during cancer cachexia [3, 31]. Our laboratory has previously utilized a systemic IL-6 receptor antibody to attenuate muscle mass loss during cancer cachexia [15, 47, 54]. This antibody also suppressed the IL-6 activation of muscle STAT3 and FIS-1 protein expression during cachexia, suggesting that the intracellular IL-6 signaling targets such as STAT3 may contribute to the regulation of mitochondrial fission [15, 47, 54, 55]. However, use of a STAT3 siRNA demonstrated no effect on mitochondrial dynamics proteins FIS-1 and MFN-1 in C2C12 myotubes during basal conditions [43]. Interestingly, the inhibition of basal STAT3 in C2C12 myotubes using C188-9 induced autophagosome accumulation but was not further induced in the presence of bafilomycin A1, suggesting a role for STAT3 in the regulation of autophagy/mitophagy processes [43]. Furthermore, STAT3 has also been shown to have a role in the regulation of mitochondrial function [36]. Evidence in non-muscle cells revealed that STAT3 is capable of translocating to the mitochondria and binding to complex I in the electron transport chain and negatively regulating mitochondrial function; however, its role remains unclear related to DRP-1 and FIS-1 [36]. Furthermore, RNA sequencing of constitutively active STAT3 MEF cells demonstrated a suppression of oxidative genes and an induction of glycolytic genes with no significant alteration to mitochondrial morphology or mass, suggesting that STAT3 may contribute primarily to mitochondrial function and not dynamics [56]. C2C12 myotubes during basal conditions [43]. Our current findings suggest that STAT3 activation is not necessary for the IL-6 induction of DRP-1 and FIS-1 in C2C12 myotubes and extends our previous work demonstrating that inhibition of STAT3 in a basal state did not alter FIS-1 [43]. However, our findings related to STAT3 regulation of DRP-1 and FIS-1 *in vitro* require further investigation *in vivo*. There is the possibility that muscle STAT3 signaling is regulating other processes in skeletal muscle that influence mitochondrial quality control, including function and autophagy/mitophagy [43, 57].

While mitochondrial dysfunction has been implicated as a driver of muscle wasting [18, 19, 21, 57], further study is required to establish whether mitochondrial dysfunction is a secondary consequence of muscle wasting [17, 20, 22, 50, 58, 59]. Our laboratory previously demonstrated that 2 weeks of systemic IL-6 overexpression is sufficient to induce STAT3 and suppress basal muscle protein synthesis in non-tumor-bearing mice [44]. Interestingly, these changes were not associated with changes in muscle or fat mass and indicate that IL-6/STAT3 signaling has consequences in muscle beyond the regulation of muscle mass [44]. Systemic IL-6 overexpression is also sufficient to suppress mitochondrial proteins COXIV and cytochrome C expression without muscle mass loss [5]. These findings suggest that the inflammation-induced disruption of mitochondrial quality control and the suppression of protein synthesis can precede skeletal muscle mass loss. In Lewis lung carcinoma (LLC), tumor-bearing mouse mitochondrial dysfunction and degeneration also occur prior to muscle mass loss, which provides further evidence for disrupted mitochondrial quality control being an early event in the muscle wasting process [58].

Overall, our results provide novel and mechanistic insights into the IL-6 regulation of skeletal muscle DRP-1 and FIS-1 expression. Two weeks of systemically elevated IL-6 was sufficient to induce both DRP-1 and FIS-1 expression, and IL-6 regulated their expression through gp130 signaling in skeletal muscle and cultured myotubes. Collectively, our results suggest that IL-6 signals through the gp130 receptor to activate ERK1/2 and induce DRP-1 and FIS-1 protein expression. In conclusion, we provide evidence for the regulation of muscle DRP-1 and FIS-1 expression through a gp130/ERK1/2 signaling axis. Further investigation is warranted to establish if accelerated fission is occurring to compensate for metabolic dysfunction and improve mitochondrial quality and if targeting the regulation of mitochondrial quality control processes can benefit skeletal muscle subjected to chronic systemic inflammation.

## Data Availability

The data used to support the findings of this study are available from the corresponding author upon request.

## Supplementary Materials

Supplementary Materials Supplemental Figure 1: basal inhibitor data.
