# Supplementary figures and images for: Regulation of Skeletal Muscle DRP-1 and FIS-1 Protein Expression by IL-6 Signaling
Source: Oxid Med Cell Longev. 2019 Feb 21;2019:8908457. doi: 10.1155/2019/8908457 (PMC6408992; doi:10.1155/2019/8908457)

Supplemental Figure 1. Basal Inhibitor Data.

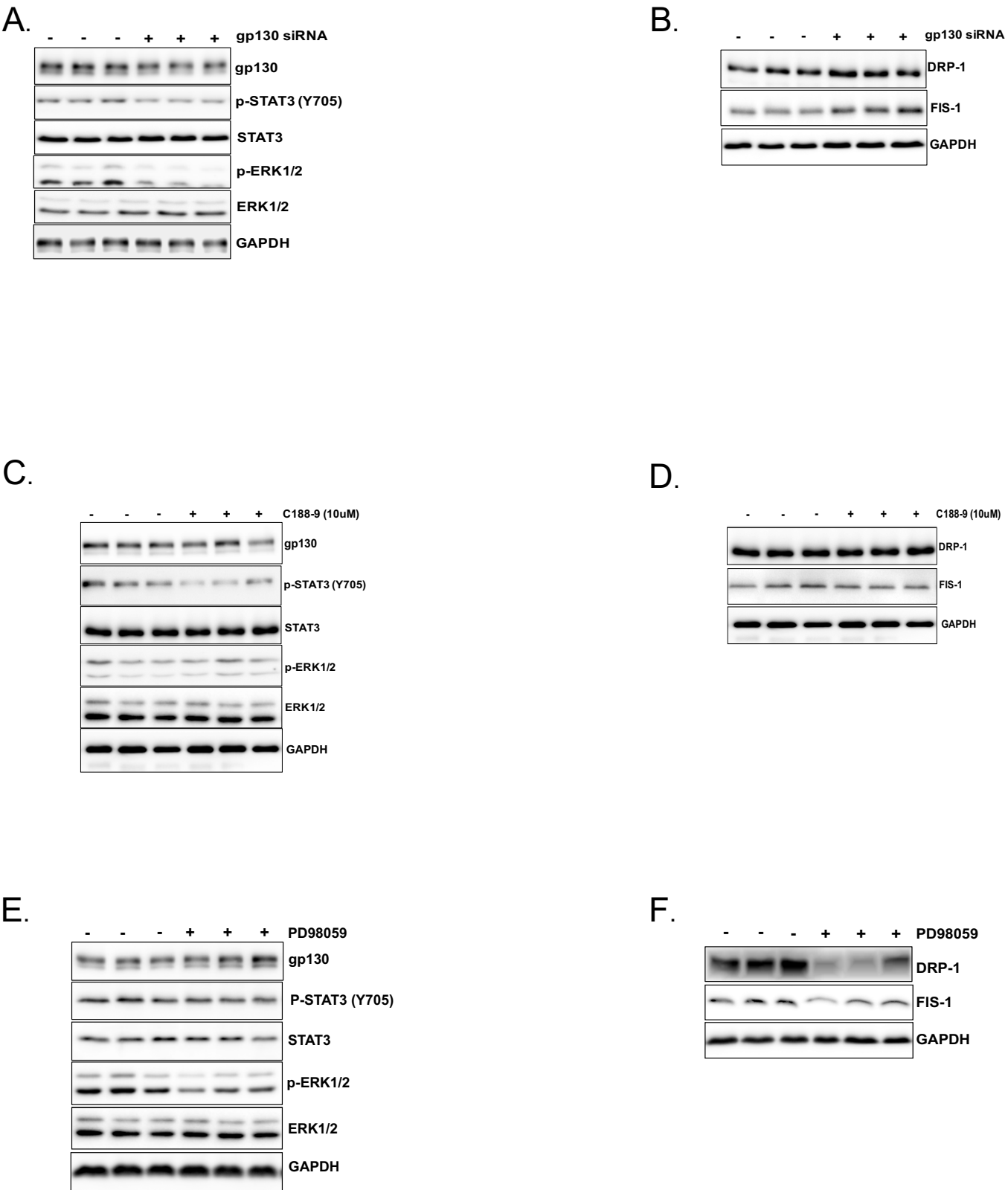

Supplement: Supplementary Materials — Supplemental Figure 1: basal inhibitor data. [file 8908457.f1.pdf]
